# Validation of the International Trauma Questionnaire—Child and Adolescent Version (ITQ-CA) in a Chinese mental health service seeking adolescent sample

**DOI:** 10.1186/s13034-022-00497-4

**Published:** 2022-08-12

**Authors:** G. W. K. Ho, H. Liu, T. Karatzias, P. Hyland, M. Cloitre, B. Lueger-Schuster, C. R. Brewin, C. Guo, X. Wang, M. Shevlin

**Affiliations:** 1 School of Nursing, The Hong Kong Polytechnic University, Hung Hom, Kowloon, Hong Kong; 2 Department of Psychiatry, Chaohu Hospital of Anhui Medical University, Hefei, China; 3 School of Mental Health and Psychological Sciences, Anhui Medical University, Hefei, China; 4 Anhui Psychiatric Center, Anhui Medical University, Hefei, China; 5 School of Health & Social Care, Edinburgh Napier University, Edinburgh, UK; 6 Rivers Centre for Traumatic Stress, NHS Lothian, Edinburgh, UK; 7 Maynooth University, Maynooth, Ireland; 8 Department of Psychiatry and Behavioral Sciences, Stanford University, Stanford, USA; 9 National Center for PTSD, Veterans Affairs Palo Alto Health Care System, Palo Alto, CA USA; 10 Department of Clinical and Health Psychology, University of Vienna, Vienna, Austria; 11 Research Department of Clinical, Educational and Health Psychology, University College London, London, UK; 12 School of Psychology, Ulster University, Derry, Northern Ireland

**Keywords:** ICD-11 PTSD, ICD-11 Complex PTSD, DSM-5 PTSD, Chinese adolescents, ITQ-CA

## Abstract

**Background:**

The International Trauma Questionnaire—Child and Adolescent version (ITQ-CA) is a self-report measure that assesses posttraumatic stress disorder (PTSD) and complex PTSD (CPTSD) based on the diagnostic formulation of the 11th version of the International Classification of Diseases (ICD-11). This study aimed to provide a Chinese translation and psychometric evaluation of the ITQ-CA using a sample of mental-health service seeking adolescents in Mainland China.

**Methods:**

The ITQ-CA was translated and back-translated from English to simplified Chinese and finalized with consensus from an expert panel. Adolescents ages 12–17 were recruited via convenience sampling from an outpatient psychiatric clinic in Mainland China. Participants completed the ITQ-CA; measures of four criterion variables (depression, anxiety, stress, adverse childhood experiences); and the PTSD Checklist for DSM-5 (PCL-5). Construct validity, concurrent validity, and comparison of PTSD caseness between ICD-11 and DSM-5 measures were assessed.

**Results:**

The final sample consisted of 111 Chinese adolescents (78% female; mean age of 15.23), all diagnosed with a major depressive disorder. Confirmatory factor analysis indicated the two-factor second-order model provided optimal fit. All criterion variables were positively and significant correlated with the six ITQ-CA symptom cluster summed scores. In the present sample, 69 participants (62.16%) met symptom criteria for ICD-PTSD or CPTSD using the ITQ-CA, and 73 participants (65.77%) met caseness for DSM-5 PTSD using the PCL-5. Rates of PTSD symptom cluster endorsement and caseness deriving from both diagnostic systems were comparable.

**Conclusions:**

The Chinese ITQ-CA has acceptable psychometric properties and confers additional benefits in identifying complex presentations of trauma-related responses in younger people seeking mental health services.

## Background

The 11th version of the International Classification of Diseases (ICD-11) [1] presents Posttraumatic Stress Disorder (PTSD) and Complex Posttraumatic Stress Disorder (CPTSD) as two distinct trauma-related disorders [2]. In ICD-11, PTSD is defined as a fear-based disorder characterized by three symptom clusters, namely (1) re-experiencing of the trauma in the here and now, (2) avoidance of traumatic reminders, and (3) a persistent sense of current threat. CPTSD includes the three PTSD symptom clusters and three additional symptom clusters that are collectively referred to as ‘Disturbances of self-organization’ (DSO). The three DSO symptom clusters include (1) affective dysregulation, (2) negative self-concept, and (3) disturbances in relationships, and may reflect more complex and severe trauma responses commonly observed among individuals who have experienced sustained or repeated forms of interpersonal trauma [2, 3]. The *International Trauma Questionnaire* (ITQ) [4] is the only self-report measure of ICD-11 PTSD and CPTSD; it is an 18-item measure that assesses 12 symptoms (2 items for each PTSD and DSO symptom cluster) and include 6 items that measure functional impairment associated with the core symptoms. The ITQ has been well-validated in both general adult populations [5–7] and in clinical or highly traumatized adult samples [8–10], but its use in children and adolescents remains limited.

The presentation and symptom structure of trauma-related disorders in younger people per ICD-11 algorithm is still under investigation, and many have highlighted a need to refine the assessment and identification of PTSD and CPTSD in children and adolescents in a manner that is in line with the new diagnostic formulation [11–13]. In response, the International Trauma Questionnaire—Child and Adolescent Version (ITQ-CA) was developed to assess PTSD and DSO symptoms in a self-report measure that is comprehensible to children and adolescents. Using the same organizing principles of the ITQ, the ITQ-CA measures the 12 core symptoms of PTSD and DSO, but includes 10 items that measure associated functional impairments. To date, the ITQ-CA has only been applied in studies with Western samples. For example, studies of Austrian foster children have reported sound factorial validity of the ITQ-CA [14]; identified maltreatment subtypes and their associations with CPTSD symptoms severity [15]; and compared diagnostic rates based on ICD-11 versus DSM-5 formulations of PTSD [16]. Studies of trauma-exposed adolescents in the general population in Lithuania also support the factorial validity of ICD-11 CPTSD using the ITQ-CA [17], and found family problems, school problems, and lack of social support as factors that differentially predicted PTSD versus CPTSD [18]. Although two recent studies of trauma-exposed adolescents in Mainland China also support the factorial validity of ICD-11 CPTSD and identified distinct symptom profiles of PTSD and CPTSD [13, 19], these studies were limited by the use of the adult version of the ITQ.

Further, no known study has investigated the reliability and validity of the ITQ-CA in psychiatric settings, which is salient given numerous studies showing children and adolescents presenting with mental health problems are more likely to have been exposed to trauma compared with those in the general population [20–22]. Indeed, children and adolescents seeking mental health services often report a history of physical, sexual, or emotional abuse, or witnessing family or community violence [23], and are at higher risk for PTSD and psychiatric comorbidity [24]. The fact that PTSD remains remarkably underdiagnosed in pediatric clinical settings [21] underscores the need for a reliable and valid measure of trauma-related disorders that can be easily administered to children and adolescents. There is currently no study of CPTSD in pediatric clinical settings. This has important diagnostic and treatment implications for trauma-exposed younger people presenting with symptoms of posttraumatic stress and/or other psychiatric disturbances.

Additionally, whether and how diagnostic rates and symptom endorsement differ across diagnostic algorithms in the child and adolescent population also warrants investigation. In contrast to ICD-11, the 5^th^ edition of the Diagnostic and Statistical Manual of Mental Disorders (DSM-5) [25] does not draw a distinction between “simple” and “complex” presentations of PTSD, and instead more broadly defines PTSD by 20 symptoms organized into four symptoms clusters: (1) re-experiencing of thoughts of traumatic event, (2) avoidance of reminders of the traumatic event, (3) persistent alterations in mood and cognitions, and (4) alterations in arousal and reactivity.[11, 26]. While the differences in diagnostic formulations of post-trauma reactions between ICD-11 and DSM-5 have been extensively discussed and researched in both general population and traumatized adult samples [27–29], little is known about how different criteria impact identification in children and adolescents, and no study has addressed this issue in a psychiatric population based on these current formulations. To our knowledge, the PTSD Checklist for DSM-5 (PCL-5) [30] is the only validated measure of DSM-5 PTSD in Chinese adolescents [31–33].

Using a sample of mental health service seeking adolescents in Mainland China, this study aimed to: (1) provide a Chinese translation of the ITQ-CA; (2) examine the factorial validity of the ITQ-CA; (3) investigate the concurrent validity of the ITQ-CA by testing its correlations with four criterion constructs (i.e. depression, anxiety, stress, exposure to adverse childhood experiences); and (4) compare PTSD caseness and symptom endorsement rates as measured by the ITQ-CA (per the diagnostic formulation of ICD-11) versus the PCL-5 (per the DSM-5).

## Methods

### Translation

This cross-sectional study provides the first translation and validation of the Chinese version of the International Trauma Questionnaire – Children and Adolescent Version (ITQ-CA). The ITQ-CA was translated and back-translated using the process suggested by Beaton, Bombardier [34]; all items were developed and modified to be comprehensible at third grade reading level. The ITQ-CA was first translated from English to simplified Chinese by a bilingual technical writer, and the content of the translated items were reviewed by an expert panel of two clinical psychologists, two mental health clinicians, and two social workers who regularly work with Chinese youths experiencing mental health problems. The panel provided comments on the clarity, understandability, and ease of answering the questions [35], and made minor adjustments to the translated items. Then, the items were back-translated to English and reviewed by the original developers of the ITQ-CA to ensure meanings were retained. Following further refinement of translated items and with consensus from the expert panel, the final Chinese ITQ-CA was pilot tested with the first 8 study participants and, without further feedback, administered to the larger sample of Chinese adolescents to assess its psychometric properties. The Chinese ITQ-CA and other language versions are available on traumameasuresglobal.com.

### Participants

Participants were recruited between January 2020 and June 2021 via convenience sampling through screening and referrals by physicians from an outpatient psychiatric clinic associated with one major university hospital in an eastern province of Mainland China. Adolescents between ages 12–17 years and in a stable condition were eligible to participate; those diagnosed with multiple psychiatric and/or comorbid physical health conditions were excluded. Participants completed paper-and-pencil surveys at the clinic after receiving endorsement from their corresponding physician, written parental/guardian consent, and with the adolescents’ assent to participate. The study was approved by the ethics committee of the second author’s affiliated institution.

### Study measures

#### ICD-11 PTSD and CPTSD

The ITQ-CA [36] is a 22-item self-report measure that assesses ICD-11 PTSD and CPTSD for people aged 7–17 years. The measure includes 6 core items of PTSD that reflect three symptom clusters: ‘Re-experiencing’ (Re1-Re2), ‘Avoidance’ (Av1-Av2), and ‘Sense of Threat’ (Th1-Th2); and 6 core items corresponding to the three symptom clusters of DSO: ‘Affective Dysregulation’ (AD1-AD2), ‘Negative Self-Concept’ (NSC1-NSC2), and ‘Disturbed Relationships’ (DR1-DR2). Respondents were asked and able to identify a target event that is currently bothering them the most, and indicated how much they were bothered by the 12 core symptoms in the past month, with responses ranging from ‘Not at all’ (0) to ‘Extremely’ (4). The internal consistency of the 12 core items in the present sample was good (α = 0.87). Functional impairment associated with PTSD and DSO symptoms were separately assessed by five additional items on interference with friendship, family relationship, schoolwork, other important life aspects, and general happiness. Probable caseness of PTSD is defined as endorsement of ‘Moderately’ (2) or above for at least one symptom in each PTSD symptom cluster; caseness of CPTSD is defined as satisfying PTSD caseness in addition to scoring ‘Moderately’ (2) or above for at least one symptom from each DSO symptom cluster. Per ICD-11, a person may receive a diagnosis of PTSD or CPTSD, but not both.

#### Criterion variables

The Chinese version of the Depression Anxiety and Stress Scale-21 (DASS-21) [37] is a 21-item self-report measure that assesses levels of depression, anxiety, and stress based on respondents’ indication of how much each statement applied to them in the past week on a 4-point Likert scale ranging from ‘Never’ (0) to ‘Almost Always’ (3). Seven items from each subscale are summed and multiplied by 2 to generate a score ranging from 0–42, with higher scores reflecting higher severity of each emotional state. The internal consistency of the DASS-21 in the current study was good (α = 0.90, 0.80, and 0.80 for depression, anxiety, and stress, respectively). Exposure to adverse childhood experiences was measured using the Chinese Adverse Childhood Experiences – International Questionnaire (ACE-IQ) [38], a 29-item self-report measure that assesses exposure to 13 ACEs, i.e. physical, sexual, emotional abuse; emotional and physical neglect; household member substance use, mental illness, incarceration; parental separation or death; domestic violence; bullying; and community and collective violence. Affirmative response to each of the 13 ACEs were summed to create an ACE score.

#### DSM-5 PTSD

The PTSD Checklist for DSM-5 (PCL-5) [30] is a 20-item self-report measure that assesses 20 symptoms of PTSD as outlined in the DSM-5 (α = 0.93 in the present sample), organized into four symptom clusters: ‘Intrusion symptoms’ (items 1–5), ‘Avoidance’ (items 6–7), ‘Negative alterations in cognition and mood’ (items 8–14), and ‘Alterations in arousal and reactivity’ (items 15–20). Respondents indicate how much they were bothered by a symptom related to the same target event they used to complete the ITQ-CA on a 5-point Likert scale ranging from ‘Not at all’ (0) to ‘Extremely’ (4). Probable caseness of PTSD is determined by endorsing symptoms at ‘Moderately’ (2) or above for at least one ‘Intrusion’ and ‘Avoidance’ symptom, and two ‘Negative alterations in cognition and mood’ and ‘Alterations in arousal and reactivity’ symptoms. The validity of the Chinese version of the PCL-5 is supported by prior studies of trauma-exposed adolescents in Mainland China [31–33], and is the only available DSM-5 PTSD measure that has been validated for use in the Chinese adolescent population.

### Data analysis

The latent structure of the ITQ-CA was tested using confirmatory factor analysis (CFA) based on responses to the 12 core symptom items using the full study sample. Two factor analytic models, the correlated six-factor and two-factor second-order models, were specified and tested based on findings from a systematic review of ITQ symptom structures demonstrating that these were the most commonly supported models [39]. The correlated six-factor model is based on the ICD-11 specification of three PTSD and three DSO symptom clusters, each measured by their respective indicators. The two-factor second-order model correlated second-order factors (PTSD and DSO) to explain the covariation among the six first-order factors, with Re, Av and Th loading on the PTSD factor and AD, NSC and, DR loading on the DSO factor. For both models the error variances were uncorrelated.

Models were estimated using Mplus 7.0 [40] and robust maximum likelihood estimation (MLR) [41], which has been shown to produce correct parameter estimates, standard errors and test statistics [42]. Model fit was assessed using standard procedures: a non-significant chi-square (χ2) test; Comparative Fit Index (CFI) and Tucker Lewis Index (TLI) values greater than 0.90; Root-Mean-Square Error of Approximation with 90% confidence intervals (RMSEA 90% CI); and Standardized Root-Mean-Square Residual (SRMR) values of 0.08 or less reflect acceptable model fit. The scaled chi-square difference test [43] and Bayesian Information Criterion (BIC) were used for model comparison. A significant difference in chi-square statistics and smaller BIC value indicate a better fitting model; a BIC value difference greater than 10 is considered a ‘significant’ difference [44]. Concurrent validity of the best fitting model was further examined by calculating the correlations between latent factors with four criterion variables – depression, anxiety, stress, and ACE score.

Finally, rates of caseness for ICD-11 and DSM-5 diagnoses and endorsement of symptom clusters in both systems were compared using McNemar tests. Concordance between the two systems was assessed using Gwet’s first-order agreement coefficient (Gwet’s AC1) [45] as it is more stable and less affected by prevalence and marginal probability than Cohen’s kappa [46]; values of 0.21–0.04 indicates fair agreement, 0.41–0.80 indicates moderate agreement, and 0.61 or above indicates substantial agreement [47]. Of note, rates for probable caseness for the ICD-11 diagnoses include those who meet ICD-11 criteria for PTSD or CPTSD, and individuals can meet criteria for PTSD or CPTSD, not both. Further, caseness rates from both systems were calculated based on symptom criteria alone (i.e. excluding functional impairment).

## Results

The final sample included 111 Chinese adolescents (78.27% female) between ages 12–17 (M = 15.23, SD = 1.44) diagnosed with a major depressive disorder. The participants reported mean scores of 24.36 (SD = 9.84) for depression, 23.78 (SD = 10.17) for anxiety, and 27.66 (SD = 9.49) for stress on the DASS-21, and were exposed to 3.01 adverse childhood experiences on average (SD = 2.47; Range = 0–10; Median = 3). The vast majority of the present sample (82.9%) reported at least one ACE.

### Construct validity

Results of the CFA showed that the fit statistics for both the correlated six-factor model and the two-factor second-order model were acceptable (see Table 1). Although the two-factor second-order model provides a closer fit to the sample data, there was no difference between the chi-square statistics between the two models (Δχ^2^ = 3.092, Δdf = 8, P = 0.928). Based on the principle of parsimony, with the second-order model having fewer parameters, the second-order model was considered the better model. Table 2 shows that all items loaded significantly and positively onto the first-order factors representative of their respective symptom cluster, ranging from 0.693 to 0.812 for the PTSD indicators and 0.455 to 0.892 for the DSO indicators. The second-order loadings for the PTSD and DSO latent variables were all high, positive and statistically significant. The correlation between the PTSD and DSO latent variables was high (*r* = 0.905, p < 0.001).

**Table 1:** Model fit statistics for alternative models of ICD-11 PTSD based on the ITQ-CA (n = 111)

Model	χ^2^	df	p	CFI	TLI	RMSEA (90% CI)	SRMR	BIC
6-factor	54.567	39	0.050	0.965	0.940	0.060 (0.000–0.312)	0.043	4067.077
2nd Order	55.663	47	0.181	0.980	0.972	0.041 (0.000–0.078)	0.045	4033.423

χ^2^, Chi-square Goodness of Fit statistic; df, degrees of freedom; p, probability value; CFI , Comparative Fit Index; TLI, Tucker Lewis Index; RMSEA (90% CI), Root-Mean-Square Error of Approximation with 90% confidence intervals; SRMR , Standardized Square Root Mean Residual; BIC, Bayesian Information Criterion

**Table 2:** Standardised Factor Loadings and Correlations for two-factor second-order model of PTSD and DSO Symptoms

Item	Re	Av	Th	AD	NSC	DR
Re1	0.693					
Re2	0.786					
Av1		0.812				
Av2		0.617				
Th1			0.748			
Th2			0.693			
AD1				0.722		
AD2				0.455		
NSC1					0.875	
NSC2					0.601	
DR1						0.892
DR2						0.924
2nd order factors						
PTSD	0.710	0.729	0.829			
DSO				0.961	0.959	0.717

All loading and correlations statistically significant (p < .001). Re1 to Th2 are the PTSD items and AD1 to DR2 are the DSO items

### Concurrent validity

The symptom cluster summed scores were all positively and significantly correlated with all criterion variables (see Table 3). Of note, correlations between stress and PTSD (0.704) and DSO (0.723) factors were of similar magnitude. Correlations between ACE score and PTSD (0.326) and DSO (0.387) were also comparable. However, anxiety more strongly correlated with PTSD (0.717) than DSO (0.565), whereas depression more strongly correlated with DSO (0.819) than PTSD (0.549).

**Table 3:** Correlations between the ICD-11 PTSD and DSO factors with criterion variables (n = 111)

	Depression	Anxiety	Stress	ACE Score
PTSD	0.549	0.717	0.704	0.326
DSO	0.819	0.565	0.723	0.387

All correlations significant (p < .001)

*ACE* Adverse childhood experiences

### Comparing ICD-11 and DSM-5 PTSD

Using the ITQ-CA, seven participants (10.14%) met caseness for ICD-11 PTSD and the remaining participants (n = 62; 89.86%) met caseness for ICD-11 CPTSD. In comparison, 73 participants (65.77%) met caseness for DSM-5 PTSD using the PCL-5. Rates of symptom cluster endorsement and caseness deriving from both diagnostic systems were comparable (see Table 4). Results indicate no statistically significant differences between symptom endorsement and caseness. Concordance between systems was significant (all *p* < *0.01*), with substantial agreement for re-experiencing and arousal /sense of threat symptoms (Gwet’s AC1 = 0.84), moderate agreement for avoidance symptoms (Gwet’s AC1 = 0.54), and fair agreement for PTSD caseness (Gwet’s AC1 = 0.36).

**Table 4:** Rates of PTSD symptom cluster endorsement and caseness by diagnostic system

	Re-experiencing	Avoidance	Arousal/Sense of Threat	Negative cognition and mood	PTSD
DSM-5	90.09	70.27	88.29	87.39	65.77
ICD-11	84.68	79.28	86.49	–	62.16
Difference	0.06^n.s^	− 0.13^n.s^	0.02^n.s^	–	0.05^n.s^
% Agreement	87.39	71.17	87.39	–	65.77
Gwet’s AC1	0.84**	0.54**	0.84**	–	0.36**

Difference = Difference in rates per cluster

^n.s.^not statistically significant

**p < 0.01

## Discussion

The present study provides the first Chinese translation and psychometric evaluation of the ITQ-CA using a mental health service seeking adolescent sample in Mainland China. This was also the first to assess ICD-11 CPTSD in a child and adolescent population with a confirmed mental health diagnosis. Overall, findings showed the Chinese ITQ-CA has sound scale reliability and validity, performs similarly in measuring caseness and symptom clusters of PTSD as compared with the PCL-5, and confers additional benefits of identifying complex presentations of trauma-related stress responses (i.e. CPTSD) in younger people.

Our sample of adolescents receiving mental health services experienced multiple adversities early in life (i.e. exposed to an average of 3 ACEs), and more than half (62%) met criteria for ICD-11 PTSD or CPTSD. These results corroborate previous research demonstrating that trauma-specific disorders are prevalent among children and adolescents receiving inpatient mental health services [20–22]. Importantly, probable diagnoses of both PTSD and CPTSD diagnoses were found in this population, with a greater proportion having CPTSD. This finding provides preliminary evidence that ICD-11 CPTSD may be a more common condition than ICD-11 PTSD in adolescents with a depressive disorder, and highlights the importance of establishing validated tools to effectively screen for and differentiate “simple” versus “complex” trauma responses in children and adolescents in the psychiatric setting. This is particularly salient given the need for different treatment planning and pathways for those who present with a complex trauma response [48].

CFA results showed the correlated six-factor model and the two-factor second-order model were both acceptable, but the latter provided a closer fit. This is consistent with prior studies demonstrating the second-order model provides a better representation of data sampled from clinical or highly traumatized adult populations using the ITQ [4, 9], and in trauma-exposed foster children using the ITQ-CA [12, 14]. Conversely, a study of trauma-exposed Chinese children sampled from the general population demonstrated the correlated six-factor model provided the best fit, but this study used the adult version of the ITQ [13]. Therefore, more research is needed to clarify the factorial structure of the ITQ-CA in assessing ICD-11 CPTSD in Chinese populations across different settings. Nonetheless, our CFA results showed PTSD and DSO factors significantly correlated with four criterion variables—depression, anxiety, stress, and ACE exposure, which are in line with the existing literature. In particular, we found anxiety was associated more strongly with PTSD than DSO, whereas depression was associated more strongly with DSO than PTSD. This pattern has been consistently observed in prior research [49, 50] and further supports the concurrent validity of the ITQ-CA.

Last, our comparison of rates of PTSD/CPTSD caseness and symptom endorsement based on ICD-11 and DSM-5 formulations showed the ITQ-CA performed similarly to the PCL-5. The only previous study of PTSD in treatment-seeking children and adolescents found that rates of DSM-IV PTSD were significantly higher than the reported ICD-11 PTSD, but this did not use an ICD-11 based measure [51]. In particular, we found substantial agreement in reports of “Re-experiencing” symptoms. This contradicts the results of a study of Austrian foster children [16] and a study of disaster-exposed children [52] that found lower endorsement of ‘Re-experiencing’ symptoms using the more stringent ICD-11 criteria as compared with the DSM-5. Therefore, our findings, at least in the context of Chinese adolescents with a comorbid mental health condition, do not support the concern that ICD-11 PTSD criteria may lead to underreporting of PTSD in children [53]. It is possible that the high levels of depression, anxiety, and stress in children and adolescents seeking mental health services (as observed in the present sample) translate to higher symptom endorsement of PTSD/CPTSD, thus meeting ICD-11 PTSD/CPTSD symptom criteria despite its greater specificity as compared with the DSM-5.

Several study limitations are noted. First, generalizability of study findings may be limited due to the relatively small sample recruited from one outpatient psychiatric clinic in Mainland China. The sample size may also have been too small to adequately power the study. However, with high factor loadings the degree to which the study is underpowered may not be too great. Nonetheless, our results require replication using larger samples from other psychiatric settings or the general population. Future studies should also examine potential confounding variables, such as length of diagnosis, psychiatric treatments received, and other sociodemographic characteristics. Second, the age range of our participants was restricted to 12–17, thus further testing with younger children is warranted given the ITQ-CA was designed to assess ICD-11 CPTSD in children as young as age 9. Third, we acknowledge the PCL-5 is an adult measure of PTSD even though it is the only available measure of DSM-5 PTSD validated for use in the Chinese adolescent population. We also asked participants to identify a target event that they were most bothered by instead of ascertaining exposure to a DSM-5 Criterion A stressor. Therefore, our comparison of ICD-11 and DSM-5 PTSD should be interpreted with caution. Last, social desirability and recall bias may have affected our findings since data were collected via self-report measures in the clinical setting.

## Conclusions

The present study supports the factorial validity of ICD-11 CPTSD and use of the ITQ-CA to measure PTSD and DSO symptoms in Chinese adolescents seeking mental health services. The ITQ-CA performed similarly in measuring caseness and symptom clusters of PTSD and CPTSD as compared with the PCL-5, and conferred additional benefits in identifying complex presentations of trauma-related stress responses (i.e. CPTSD) in younger people.

## Data Availability

The dataset used and/or analyzed during the current study are available from the corresponding author on reasonable request.

## Abbreviations

ITQ-CA: The International Trauma Questionnaire – Child and Adolescent version
ICD-11: 11^Th^ version of the International Classification of Diseases
PTSD: Posttraumatic stress disorder
CPTSD: Complex posttraumatic stress disorder
